# Epidemiological impacts of nonpharmaceutical interventions are modulated by immunity exposure trade offs

**DOI:** 10.1038/s43856-026-01492-y

**Published:** 2026-05-01

**Authors:** Chadi M. Saad-Roy, Bjarke Frost Nielsen, Margaret L. Lind, Caroline E. Wagner, Arne Traulsen, C. Jessica E. Metcalf, Mike Boots, Derek A. T. Cummings, Bryan T. Grenfell

**Affiliations:** 1 Miller Institute for Basic Research in Science, University of California, Berkeley, CA USA; 2 Department of Integrative Biology, University of California, Berkeley, CA USA; 3 Department of Mathematics, University of British Columbia, Vancouver, BC Canada; 4 Department of Microbiology and Immunology, University of British Columbia, Vancouver, BC Canada; 5 Biodiversity Research Centre, University of British Columbia, Vancouver, BC Canada; 6 Department of Ecology and Evolutionary Biology, Princeton University, Princeton, NJ USA; 7 Niels Bohr Institute, University of Copenhagen, Copenhagen, Denmark; 8 PandemiX Center, Roskilde University, Roskilde, Denmark; 9 Department of Epidemiology of Microbial Diseases, Yale School of Public Health, New Haven, CT USA; 10 Department of Epidemiology, Boston University School of Public Health, Boston, MA USA; 11 Department of Bioengineering, McGill University, Montreal, QC Canada; 12 Max Planck Institute for Evolutionary Biology, Plön, Germany; 13 School of Public and International Affairs, Princeton University, Princeton, NJ USA; 14 Department of Biosciences, University of Exeter, Exeter, UK; 15 Department of Biology, University of Florida, Gainesville, FL USA; 16 Emerging Pathogens Institute, University of Florida, Gainesville, FL USA

**Keywords:** Viral infection, Virus-host interactions

## Abstract

**Background:**

The COVID-19 pandemic has illustrated how nonpharmaceutical interventions (NPIs), such as mask-wearing, social distancing, and purifying air, can successfully mitigate transmission and reduce infections in the short term. However, the longer-term implications of these interventions on infection levels are less clear. In tandem, recent observational evidence suggests that the relative susceptibility of partially immune individuals to infection may be dose-dependent, i.e., higher exposures are more likely to result in (re)infection in individuals with prior immunity from infection or vaccination.

**Methods:**

To examine this question, we use mechanistic immuno-epidemiological models to determine the equilibrium infection levels with NPI-induced reductions in transmission in the presence of this dose-dependency.

**Results:**

We find that NPIs can successfully decrease the number of infections at endemicity, even in high transmission scenarios. We also show that this effect is heightened by vaccination, especially if a durable, broadly-protective (i.e., broad antigen specificity) vaccine is deployed. Finally, we find that NPI-induced declines in infection levels are strongly magnified if the characteristics of subsequent infections, such as transmissibility or duration, are also dose-dependent.

**Conclusions:**

Overall, our results suggest that the long-term usage of NPIs could successfully reduce infections especially where immunity-exposure trade-offs apply due to dose-dependency, and illustrate the urgent need to characterize the underlying relationships between exposure and host immune responses.

## Plain language summary

The COVID-19 pandemic underscored the importance of nonpharmaceutical interventions (NPIs) on short-term infection levels and the impacts of host immunity on epidemic dynamics. Empirical findings suggest that the relative susceptibility of individuals previously infected or vaccinated may hinge on the dose of exposure. Thus, NPIs that decrease viral exposure may have unexplored benefits. With an immuno-epidemiological model, we find that NPIs can successfully reduce endemic infection levels in the presence of such dose-dependence, even in high-transmission settings. If this relationship between exposure and relative susceptibility extends to the duration of subsequent infections or their relative transmissibility, we show that this reduction is magnified. Finally, we find that vaccination also strengthens this effect, which highlights the importance of broad-scale vaccine uptake.

## Abstract (web summary)

Saad-Roy et al. used theoretical models to examine the implications of nonpharmaceutical interventions (NPIs) on endemic infection levels when there is dose-dependent immunity. Overall, they find that dose-dependent immunity can amplify NPI-driven reductions in endemic infections, including leading to sizable reductions in settings where NPIs do not have an appreciable effect otherwise.

## Introduction

Since 2020, the SARS-CoV-2 pandemic has caused tremendous morbidity and mortality worldwide^1^. Initial control efforts were dominated by the implementation of nonpharmaceutical interventions (NPIs), such as social distancing and mask wearing, that aimed to reduce transmission and consequently decrease the level of circulating SARS-CoV-2 infections. In tandem, such NPIs deployed to mitigate SARS-CoV-2 transmission also controlled other seasonal respiratory pathogens^2^, which led to a buildup of susceptibles^3^. To decrease this population-level susceptibility and achieve pathogen control via community immunity (without infection), transmission-blocking vaccines are necessary (see, e.g., refs. ^4–7^).

A number of vaccines for SARS-CoV-2 were successfully developed and deployed across the world (e.g., refs. ^8,9^). However, while these existing formulations are very effective against severe disease, they are only transiently protective against transmission. In the absence of robust transmission-blocking vaccines, population-level immunity cannot be reached via vaccination alone. Instead, as we have seen, the combination of vaccination and recovery from prior infection has led to widespread population-level immunity against severe disease. While this has decreased the rate of case hospitalization, and we have now consequently relaxed mandates for NPI adherence, SARS-CoV-2 still circulates and strongly impacts at-risk populations (such as the elderly) (see e.g., ref. ^10^).

However, the potential long-term immuno-epidemiological outcomes of NPIs for endemic infections remain uncertain. Assuming that individuals are either fully immune for life or eventually return to complete susceptibility, recent work has shown that decreases in transmission rates can lead to lower endemic levels (and potential control) for pathogens with low basic reproduction numbers^11^. On the other hand, for pathogens with high basic reproduction numbers, decreases in transmission lead to an initial (short) transient period of control but negligibly affect the longer-term equilibrium level of infections^11^. Furthermore, from a clinical standpoint, recent work has shown that COVID-19 severity is not likely linked with innoculum dose^12^. However, emerging evidence suggests that higher individual exposures to SARS-CoV-2 can overcome existing immunity (derived from either infection or vaccination) and lead to infection, whereas lower exposures do not^13^. Thus, understanding the impacts of such immunity-exposure trade-offs is crucial, especially in the context of NPIs. In particular, this could inform various aspects of ongoing policy, especially those deployed to decrease exposure magnitude, such as mask-wearing or cleaner-air initiatives (e.g., with Far-UVC^14^).

While classical epidemiological models assume that individuals either have lifelong immunity or eventually become fully susceptible after a period of complete immunity has waned (see e.g., ref. ^4^), individuals may instead have partial susceptibility to infection after the waning of complete immunity. Furthermore, once infected, these individuals may have infections that are less transmissible. To capture this ’buffered susceptibility’, we previously used the SIR(S) framework^7,15^ to show that the relative susceptibility to secondary infection (after infection-derived or vaccinal immunity has waned) is a key determinant of potential medium-term SARS-CoV-2 trajectories^7^. Here, ‘secondary’ infection is defined as an infection that occurs after a first infection or after vaccination (thus, the individual experiencing such an infection is not immunologically naive). We then subsequently refined this model framework to investigate the implications of vaccine dosing regimes^16^, vaccine nationalism^17^, the potential accumulation of immunity combined with chronic disease^18^, and the impacts of waning severity-blocking immunity^19^. The feedbacks between population-level immunity to SARS-CoV-2 and infection were studied in a number of influential papers since 2020 (see ref. ^20^ for an excellent review and references therein). For example, some have focused on how and when to social distance^21^, and on the COVID-19 pandemic in Canada^22,23^. Others have used models to examine vaccine sharing from a data-driven perspective^24^, or to study the implications of vaccines for community immunity^5^.

In this paper, we extend the SIR(S) mathematical framework^7,15^ to titrate the impacts of an immunity-exposure trade-off on the dynamics of circulating pathogens. Using our model, we then determine the qualitative effects on equilibrium levels of infections if exposures in previously infected or vaccinated individuals are dose-dependent, i.e., if decreases in exposure magnitude also decrease the likelihood of infection among individuals with immunity due to infection or vaccination. We also examine the impact of additional (protective) effects of host immune responses that could also depend on the degree of exposure, such as the duration or transmissibility of a secondary infection. Finally, we investigate the combined effects of vaccination with NPIs.

## Methods

**Fig. 1:**
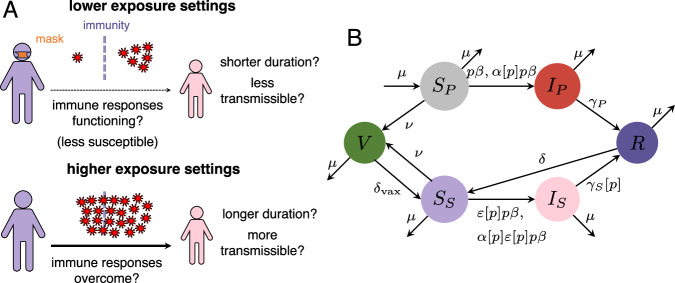
Model framework to capture an exposure-immunity trade-off. **A** Illustrative schematic of this trade-off, where lower exposure settings can reduce the relative susceptibility to infection in (potentially) partially immune individuals, but higher exposure settings overcome existing immunity and lead to infection. The characteristics of these two kinds of subsequent infections could be different in duration and transmissibility. **B** Flow diagram of the modified SIR(S) model with vaccination^7^. As in ref. ^7^, the following parameters are defined as follows: *μ* is the birth/death rate, *β* is the transmission rate, *ν* is the vaccination rate, *δ* is the infection-derived immunity waning rate, *δ*_vax_ is the vaccinal immunity waning rate, *ε* is the relative susceptibility to a secondary infection, *α* is the relative transmissibility of a secondary infection (note that 0≤*ε*≤1 and 0≤*α*≤1). In contrast to ref. ^7^, the recovery rates *γ*_*P*_ and *γ*_*S*_ from primary (*I*_*P*_) and secondary (*I*_*S*_) infections are not necessarily the same, and *p* is such that *pβ* is the NPI-induced transmission rate. **B** is adapted from ref. ^7^.

To examine the implications of an immunity-exposure trade-off, we build upon the SIR(S) model with random vaccination^7,15^. As in ref. ^7^, *S*_*P*_ denotes individuals that are fully susceptible and have never been infected nor vaccinated, *I*_*P*_ denotes individuals with a primary infection (i.e., from the fully susceptible pool *S*_*P*_), *R* denotes individuals that are fully immune to infection following recovery, *V* denotes individuals that are fully immune following vaccination, *S*_*S*_ denotes those individuals that are (potentially partially) susceptible after their immunity has waned, and *I*_*S*_ denotes individuals with a secondary infection (i.e., from the secondary susceptible pool *S*_*S*_). Here, the model does not explicitly differentiate whether these secondary infections are the result of antigenic drift (a viral property) or actual host immune waning (a host property). As such, if, after an initial infection, a host can get infected again, either by the same pathogen or a novel variant, then this would be classified as a “secondary infection”. However, while the simplest scenarios involving antigenic evolution are covered by our model, full variant dynamics in the presence of dose-dependent immunity would require refined, more complex models. Note that (as in ref. ^7^) we assume that vaccination is random and that it only affects susceptible individuals, i.e., those in *S*_*P*_ or *S*_*S*_ (see caption of Fig. 1B for definition of all model parameters).

First, since secondary infections may have a different average duration than primary infections, we include a potentially different rate of recovery from a secondary infection, i.e., *γ*_*P*_ and *γ*_*S*_ are the recovery rates from primary and secondary infections, respectively. Second, we incorporate a parameter *p* that denotes the relative transmission rate with ongoing interventions (i.e., *pβ* is the NPI-induced transmission rate). Finally, since the NPI-induced transmission rate is *pβ*, it follows that the relative exposure is determined by *p*. We therefore assume that the relative susceptibility to infection in previously-immune individuals, the relative transmissibility of an infection by previously-immune individuals, and the recovery rate from a secondary infection are (potentially) functions of *p*, i.e., *ε*[*p*], *α*[*p*], and *γ*_*S*_[*p*], respectively. Figure 1B illustrates the flow diagram of our model, and other parameter definitions are given in the caption.

Therefore, the model equations are

1a $$\frac{d{S}_{P}}{dt}=\mu -p\beta {S}_{P}({I}_{P}+\alpha {I}_{S})-(\mu +\nu ){S}_{P},$$

1b $$\frac{d{I}_{P}}{dt}=p\beta {S}_{P}({I}_{P}+\alpha {I}_{S})-(\mu +{\gamma }_{P}){I}_{P},$$

1c $$\frac{dR}{dt}={\gamma }_{P}{I}_{P}+{\gamma }_{S}{I}_{S}-(\delta +\mu )R,$$

1d $$\frac{dV}{dt}=\nu ({S}_{P}+{S}_{S})-({\delta }_{{{\rm{vax}}}}+\mu )V,$$

1e $$\frac{d{S}_{S}}{dt}=\delta R+{\delta }_{{{\rm{vax}}}}V-\varepsilon p\beta {S}_{S}({I}_{P}+\alpha {I}_{S})-(\mu +\nu ){S}_{S},$$

1f $$\frac{d{I}_{S}}{dt}=\varepsilon p\beta {S}_{S}({I}_{P}+\alpha {I}_{S})-({\gamma }_{S}+\mu ){I}_{S}.$$

In the Supplementary Information, we use the next-generation matrix approach^25,26^ to show that the associated basic reproduction number of this model is

2 $${{{\mathcal{R}}}}_{0}=\frac{p\beta }{{\gamma }_{P}+\mu }\frac{\mu }{\mu +\nu }+\varepsilon [p]\alpha [p]\frac{p\beta }{{\gamma }_{S}[p]+\mu }\frac{\nu }{\mu +\nu }\frac{{\delta }_{{{\rm{vax}}}}}{{\delta }_{{{\rm{vax}}}}+\nu +\mu }.$$

We also prove that if the duration of a secondary infection does not exceed that of a primary infection, then there is a unique equilibrium in our model and ${{{\mathcal{R}}}}_{0}$ is a sharp threshold, i.e., there is only the disease-free equilibrium if ${{{\mathcal{R}}}}_{0} < 1$ (Theorem 1, Supplementary Information). The expressions for the endemic equilibrium of each fraction are given in the proof of Theorem 1 (Supplementary Information). In all that follows, we use these expressions to numerically obtain the values of endemic infection levels (i.e., ${\widehat{I}}_{P}+{\widehat{I}}_{S}$) for given parameter values.

## Results

### Baseline model

In line with real-world evidence for SARS-CoV-2, we first assume a relatively short duration of complete infection-derived or vaccinal immunity to infection. Furthermore, to allow for proper comparison with prior work on SIR/SIRS models^11^, we initially assume that primary and secondary infections are indistinguishable (i.e., same recovery rate and relative transmissibility, see ref. ^7^). Thus, if the susceptibility to primary and secondary infection is identical (*ε* = 1), our model is equivalent to an SIRS model. On the other hand, if the relative susceptibility to secondary infections is zero, our model reduces to an SIR model. We begin with the assumption that only the relative susceptibility to infection in previously-exposed individuals is dose-dependent.

**Fig. 2:**
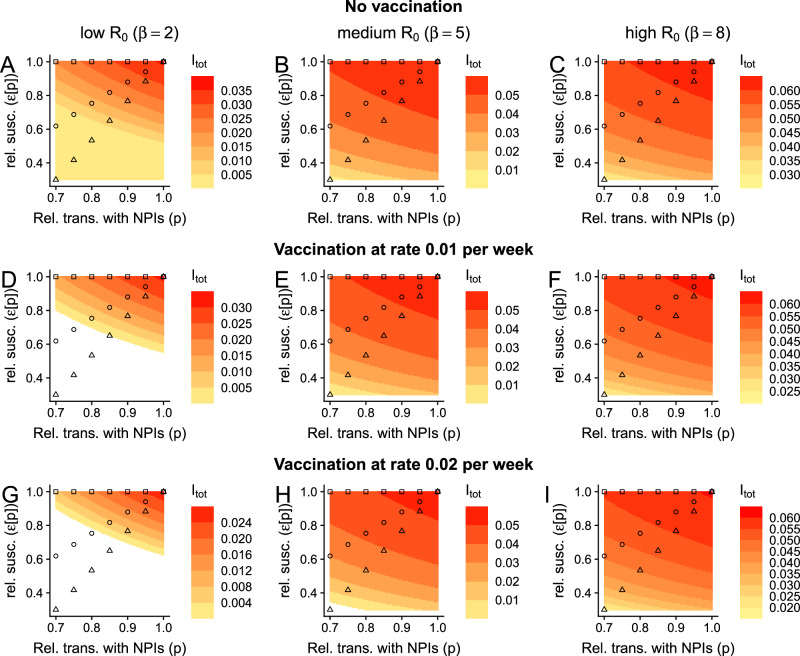
Impacts of a transmission-exposure trade-off on endemic infection levels. Each panel depicts the total fraction of the population that is infected, i.e., *I*_tot_ = *I*_*P*_ + *I*_*S*_ at equilibrium as a function of the NPI-induced relative transmission rate and the relative susceptibility to infection in previously exposed individuals after complete immunity has waned. **A**–**C** depict scenarios with no vaccination (*ν* = 0), whereas **D**–**F** and **G**–**I** have vaccination at rate *ν* = 0.01 and *ν* = 0.02, respectively. Epidemiological outcomes in low-, medium-, and high-transmission settings are presented in (**A**, **D**, **G**), (**B**, **E**, **H**), and (**C**, **F**, **I**), respectively. The square, circle, and triangle markers represent, respectively, scenarios where there is no, a moderate, or a strong relationship between exposure and susceptibility to infection in previously exposed individuals. Throughout all panels, white regions are where *I*_tot_ = 0, and other parameters are *μ* = 0.02 per year, $\frac{1}{\delta }=\frac{1}{{\delta }_{{{\rm{vax}}}}}=0.25$ years, *γ*_*P*_ = *γ*_*S*_ = 1 per week, and *α* = 1. For the illustrative relationships between *ε* and *p*, depicted by squares, triangles, and circles, we use the functions *ε*_□_[*p*] = 1, ${\varepsilon }_{\bigtriangleup }[p]=\frac{7}{3}p-\frac{4}{3}$, and ${\varepsilon }_{\circ }[p]=\frac{7}{3}{p}^{\frac{1}{2}}-\frac{4}{3}$, respectively, for 0.7 ≤ *p* ≤ 1.

In Fig. 2A–C, we examine potential endemic infection levels for low, medium, and high transmission scenarios, respectively, each as a function of both the relative transmission rate *p* and relative susceptibility to secondary infection *ε*. Since the quantitative details of the trade-off between immunity and exposure are unresolved, this approach allows us to visualize the effect of any functional dependence between these parameters. As examples, we illustratively represent three potential relationships on the heatmap for each transmission scenario. The first (in square symbols) denotes a setting where *ε* and *p* are independent, and this recapitulates previous modeling work with the SIRS model^11^. We then depict a more modest and a more pronounced immunity-exposure trade-off, in circle and triangular symbols, respectively. For these scenarios, a decrease in the NPI-induced relative transmission rate (driven by higher adherence or more effective NPIs) leads to a decrease in relative susceptibility to infection in previously-exposed individuals. Note that these relationships between *p* and *ε* are identical throughout and are independent of the endemic level of infections. Instead, we use these qualitative examples to determine the effects of a dependence between *p* and *ε* on endemic infection levels.

In low-transmission settings, without vaccination, and without an immunity-exposure trade-off, decreasing transmission has an appreciable effect on the endemic level of infection (Fig. 2A, square symbols), and this was thoroughly explored in previous work^11^. However, our analysis in Fig. 2A shows that a trade-off between immunity and exposure magnifies the dependence of endemic infection levels on the NPI-induced relative transmission rate (Fig. 2A, compare symbols). Thus, immune-driven dose-dependent susceptibility to infection can result in major quantitative differences in the number of infections at equilibrium, and the relative effectiveness of NPIs to reduce long-term burden will crucially hinge on the shape of this trade-off.

In high-transmission settings and without a trade-off between immunity and exposure, NPIs short of the elimination threshold have very limited effects in the long-term^11^. However, with such a trade-off, we find that important qualitative differences in outcomes emerge. In particular, this trade-off introduces a substantive decrease in equilibrium infection levels as the NPI-induced relative transmission rate increases (Fig. 2C). This dependence is rapidly magnified as the trade-off becomes more pronounced, i.e., as the relative susceptibility to infection in previously-exposed individuals decreases more rapidly with increasing NPIs.

Across transmission scenarios and irrespective of any underlying trade-off between immunity and exposure, we also find that vaccination decreases equilibrium levels of infections (Fig. 2, which echoes ref. ^11^). Furthermore, when there is a trade-off, vaccination accentuates the effects of dose-dependent infections (compare rows of Fig. 2). Thus, this result highlights the benefits of ongoing vaccination in conjunction with NPIs, and of increasing vaccine uptake (e.g., by decreasing hesitancy^27,28^).

Note that while we specify that *ε* could be a function of *p*, and we schematically illustrate possible relationships for specific examples (given by squares, circles, and triangles), our results, as presented in these heatmaps, are agnostic of specific relationships. Instead, the effect of any functional dependence of *ε* on *p* can be determined from the representations in Fig. 2. This approach is particularly powerful because we do not yet know the relationship between these parameters, and therefore motivates the importance of data collection to accurately measure this relationship.

### Impacts of broadly-protective vaccines

**Fig. 3:**
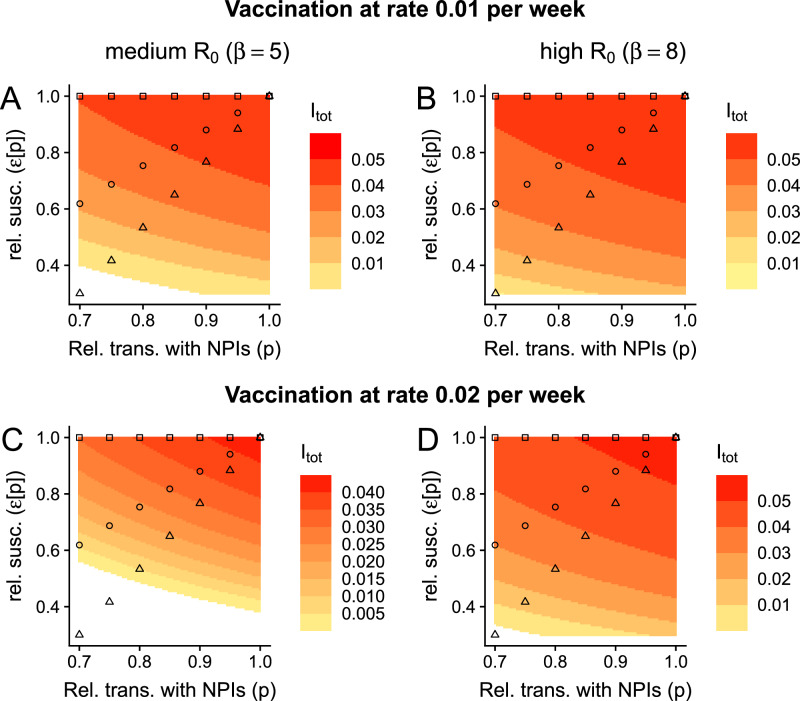
Effects of a broadly-protective vaccine on the implications of a trade-off between immunity and exposure. Here, we assume that a vaccine imparts complete immunity for an average of a year, i.e., $\frac{1}{{\delta }_{{{\rm{vax}}}}}=1$ year. **A**, **C** denote a medium-transmission setting, whereas **B**, **D** denote a high-transmission setting. Furthermore, in **A**, **B**, *ν* = 0.01 per week, whereas *ν* = 0.02 per week in (**C**, **D**). All other details and parameters are as in Fig. 2.

Since infections and current SARS-CoV-2 vaccines likely provide very transient protection against infection, we also assume that the average duration of complete immunity is short-lasting. Furthermore, to enable comparisons between our model with those for the SIRS model with vaccination^11^ (i.e., so that our model reduces to the SIRS case when *ε* = 1), we have assumed that the duration of complete vaccinal immunity is identical to that of infection-induced immunity. However, there are large initiatives currently underway to develop broadly-protective SARS-CoV-2 vaccines^29–31^, which would hopefully provide longer-lasting substantial protection against infection (by conferring relevant protection against a larger number of possible variants). In Fig. 3, we examine the effects of a vaccine that completely protects an individual from infection for a year on average. We surprisingly find that such a vaccine accentuates the effects of a trade-off between exposure and relative susceptibility (compare Fig. 3A–D with Fig. 2E, F, H, I, respectively). In particular, in these settings and as *p* decreases, infection levels have a more pronounced decline. Furthermore, in these medium- and high-transmission settings, high vaccination rates combined with NPIs can result in local elimination. If complete vaccinal immunity lasts instead 2 years on average, these features are further amplified (Fig. S1).

### Lower exposure decreases duration of infection in previously-immune individuals

**Fig. 4:**
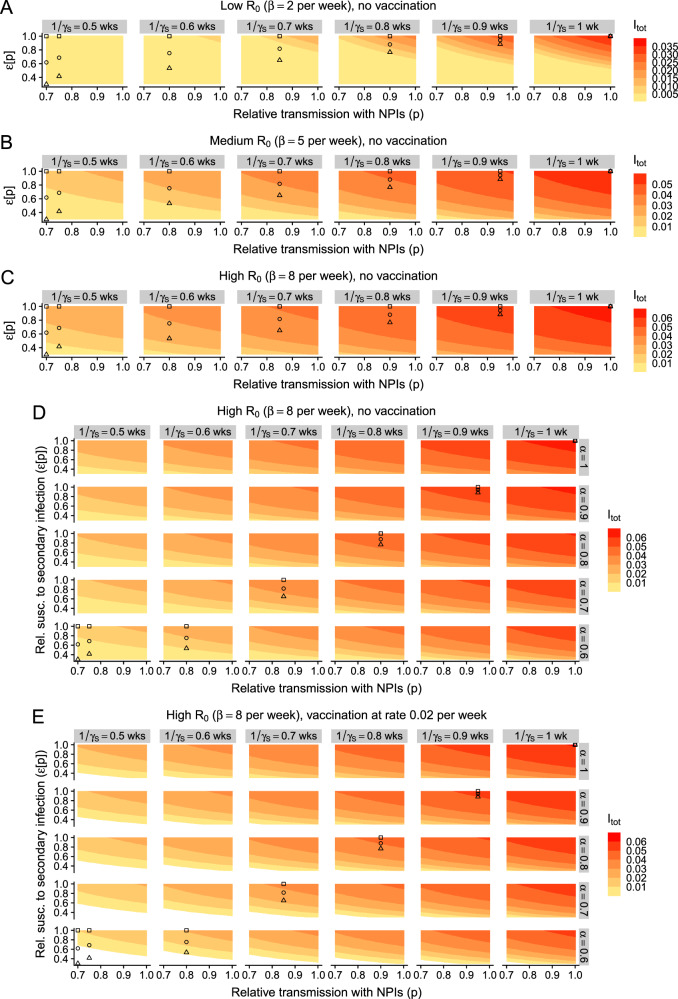
Effect of dose-dependent duration of infection (in previously exposed individuals) on the implications of NPI-induced reductions in exposure. **A** Low-transmission setting with no vaccination, **B** medium-transmission setting with no vaccination, **C** high-transmission setting with no vaccination. **D**, **E**: Outcomes in high-transmission settings with a combination of dose-dependent relative susceptibility to secondary infection *ε*, duration of secondary infection $\frac{1}{{\gamma }_{S}}$, and relative transmissibility of secondary infection *α*. Additionally, **D**, **E** depict scenarios with no vaccination and vaccination at a rate of *ν* = 0.02 per week, respectively. All details and other parameters are as in Fig. 2.

So far, we have assumed that only the relative susceptibility to infection of previously-exposed individuals is dose-dependent, and that the characteristics of an infection in these individuals are identical to those in never-exposed infected individuals. In particular, and as in prior work, we have taken secondary infections to have the same transmissibility and duration as that of primary infections (i.e., *α* = 1 and *γ*_*S*_ = *γ*_*P*_, respectively, see e.g., refs. ^7,16^). However, in previously exposed individuals, prior immunity could act to decrease the duration of infection or reduce its transmissibility, especially in lower-exposure settings. Thus, these features could also be dose-dependent, i.e., decrease with lower exposure. In Fig. 4A–C, we explore the impact of dose-dependent duration of infections in previously exposed individuals. As in Figs. 2 and 3, we examine equilibrium infection levels for a range of NPI-induced relative transmission rates, relative susceptibility rates, and durations of secondary infection. In Fig. 4, we also present the illustrative relationships between immunity and exposure from Fig. 2 and expand these so that the duration of secondary infections decreases with lower exposure. To allow for appropriate comparison between scenarios, we assume that these illustrative schematics (i.e., shown with the squares, circles, and triangles) depict the same relationship between duration of secondary infection i.e., $\left(\frac{1}{{\gamma }_{S}}\right)$ and *p*.

In the absence of a decrease in relative susceptibility to secondary infection, we also find that a trade-off between exposure and the duration of secondary infection has a qualitatively similar effect on the equilibrium number of infections (Fig. 4A–C, square symbols). In low-transmission settings, moderate reductions in the duration of secondary infection cause a substantive reduction in infection levels (Fig. 4A). While this effect is dampened as transmission increases, it nevertheless persists (compare Fig. 4A–C). Importantly, such a reduction in duration of secondary infections even leads to more marked reductions in infection levels than if exposure only affects relative susceptibility to secondary infection. Thus, it is imperative to quantify the duration of secondary infections as a function of exposure levels.

Overall, when relative susceptibility decreases as exposure decreases, we find that a dose-dependent duration of secondary infection heightens our previously observed effects (compare plots within each panel of Fig. 4A–C from right to left). In these settings, vaccination also functions to augment the potential effects of a trade-off between immunity and exposure (Figs. S2 and S3). Finally, these are then themselves further magnified if the duration of complete vaccinal immunity is increased via a broadly-protective vaccine (Figs. S4, S5 and S7, S8).

### Lower exposure decreases relative transmissibility of infections in priorly-immune individuals

While prior immunity could decrease the duration of secondary infections, it may also act to reduce the relative transmissibility of these infections. In Fig. 4D, E, we explore this in detail for high-transmission settings. If the relative transmissibility of secondary infections is also dose-dependent, the effects of any trade-off between immunity and exposure are again further amplified. However, the decrease in infection levels that is induced via a decrease in relative transmissibility alone appears to be more moderate than those due to a decrease in relative susceptibility or a decrease in duration of secondary infection (compare rows in Fig. 4D with the top panel in each column, and the top row with the rightmost column in Fig. 4D, respectively). In line with our previous results, vaccination accentuates any trade-off between immunity and exposure (compare Fig. 4E with 4D), and this is boosted further if a vaccine confers durable complete immunity (Figs. S6 and S9).

### Summary

**Fig. 5:**
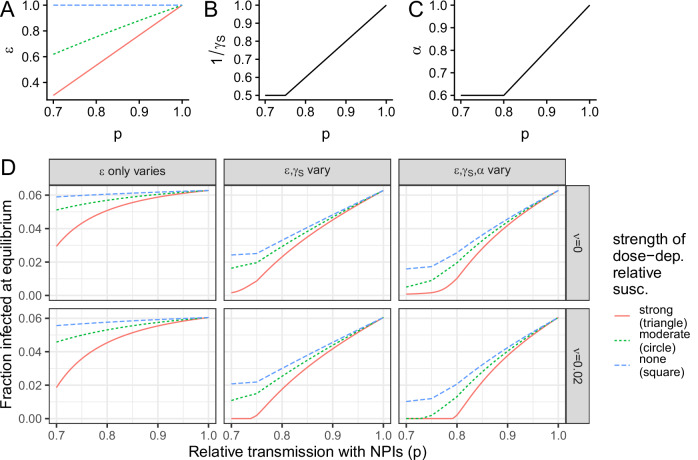
Summary illustrative schematic for the impacts of a dose-dependence on secondary infection characteristics. **A** Relationship between relative susceptibility (*ε*) and relative transmission with NPIs (*p*), for strong, moderate, and no dose-dependence, which are as the relationship depicted in Figs. 2 and 3 by circles, triangles, and squares, respectively. Relationship between the average duration (**B**) or the relative transmissibility (**C**) of a secondary infection as a function of the relative transmission with NPIs (*p*), as taken for all three illustrative relationships (i.e., circles, triangles, and squares) depicted in the relevant panels of Fig. 3. **D** Endemic infection levels in different settings, including which parameters are dose-dependent (*columns*) and whether there is vaccination (*row*).

To summarize our results, we present in Fig. 5 the impacts on endemic infection levels for the three illustrative trade-offs (Fig. 5A, i.e., depicted by squares, circles, and triangles in Figs. 2, 3) compounded with the potential additional dependence of the duration (Fig. 5B) and relative transmissibility (Fig. 5C) of secondary infections on dose. Even when only the relative susceptibility to secondary infections is dose-dependent, NPIs can decrease infection levels substantially, and the benefits per increase in NPI usage are nonlinear (Fig. 5D, leftmost column). If additional immune parameters are also dose-dependent, NPI usage further decreases endemic infections and can even lead to local elimination (Fig. 5D, middle and rightmost columns). Thus, this highlights the potential additional benefit of NPIs. Taken together, our results highlight the urgent need to characterize the shape of immunity-transmission trade-offs to guide public health recommendations.

## Discussion and conclusions

As we have seen during the SARS-CoV-2 pandemic, the implementation of NPIs early in an outbreak clearly reduces ongoing transmission and reduces infection levels^2^. In parallel, and again driven by a decrease in transmission opportunities, such measures also transiently mitigate circulating infections, e.g., influenza viruses, respiratory syncytial virus, or enteroviruses^11,32^. While the impact of NPIs on early epidemic trajectories is governed by the magnitude of the basic reproduction number, NPI-induced reductions in transmission eventually result in an accumulation of susceptibles^11^, many of whom were previously infected or vaccinated. Our findings illustrate that long-term population-level outcomes also hinge on the characteristics of these previously-immune susceptibles, such as their likelihood of infection relative to those never-infected. In particular, we find that dose-dependent infection among partially immune individuals can lead to a marked reduction in total infection levels at equilibrium, even for pathogens with high basic reproduction numbers. Furthermore, we show that this effect depends crucially on the strength of the underlying trade-off between immunity and exposure, and that it can be enhanced by vaccination, particularly with a durable vaccine. We also find that the beneficial effects of long-term NPI usage compound if either the duration or transmissibility of secondary infections also depend on exposure level. Our results, therefore, suggest that models that do not include this relationship between immunity and exposure may underestimate the effect of NPIs or vaccination on endemic infection levels.

In high transmission settings, the substantive qualitative effects of an immunity-exposure trade-off have multiple implications. First, these findings imply that long-term NPI usage may be a suitable approach for infection control, which contrasts to findings of previous work when ${{{\mathcal{R}}}}_{0}$ is high^11^. Since NPI adherence is usually shaped by a combination of individual and societal approaches, understanding NPI-specific drivers of adherence will be crucial. For example, certain interventions may be perceived as more costly from an individual perspective, and individual decision-making could limit population-level adherence (e.g., mask-wearing or social distancing, see refs. ^33–35^ for extensive discussions). In that case, top-down mandates could be beneficial to ensure necessary levels of adherence. In contrast, other mitigation measures, such as clean-air initiatives, are societally driven and may not face the same uptake barriers. In particular, there has been a resurgence of interest in Far-UVC filtration methods, and these could successfully decrease exposure^14^. Thus, such a measure may be an important method of control, especially if exposures are dose-dependent.

In the absence of empirical data, theoretical research can reveal potentially important biological effects that otherwise would be unknown. The recent work by Lind et al.^13^ has shown that an increase in the magnitude of exposure to SARS-CoV-2 could overwhelm immune responses and lead to reinfection, whereas lower exposures would not. The long-term population-level effects of this dose-dependence, especially in confluence with NPIs (and vaccination), remained unknown, and here we used theoretical approaches to resolve this qualitatively and show that long-term NPI adherence (coupled with vaccination) could successfully decrease infection levels in the presence of such dose-dependence. To make quantitative epidemiological predictions, our findings motivate a number of empirical directions. For example, immuno-epidemiological cohort studies that track infection status, serology, and vaccination (see e.g., ref. ^36^ for such a proposal) could elucidate the relationship between immunity and exposure, especially if such studies include data on exposure settings. In a different direction, our work also motivates the need to clarify within-host kinetics in previously-infected or vaccinated individuals, which could aid in disentangling the relationship between immunity and exposure.

We have also made a number of simplifying assumptions in our modeling framework that should be relaxed in future work. First, for analytical tractability and to qualitatively determine the impact of an immunity-exposure trade-off on endemic outcomes, we have omitted various types of heterogeneities, including in transmission^37–40^, or age^41^. These heterogeneities could quantitatively impact long-term immuno-epidemiology, especially if the underlying immunity-exposure trade-off is itself heterogeneous, e.g., in space, age, or uptake of NPIs. For example, if dose-dependence is only pronounced for a subgroup of the population, our findings in high-transmission settings would likely be partially buffered, and the quantitative change in infection levels reduced. However, if the average dose-dependence remains constant across the population, then determining the impact of heterogeneity on the effect we observe is an important area for future work. Ultimately, these questions should be resolved with refined models. In Remark 1 (Supplementary Information), we explore the simplest case with two non-interacting groups, and explain how our current modeling framework can capture this setting. Finally, uptake of NPIs can be heterogeneous, and there may also be assortative mixing based upon NPI adoption. The impacts of dose-dependent immunity in these settings should be examined in future work.

Second, we have also omitted the immuno-epidemiological implications of dose-dependent exposures with pathogen evolution, and this is a particularly salient area of future work^36,40^. In particular, pathogen evolution could affect the relationship between exposure and immunity, in addition to changing transmission, immunity, or the duration of infection. Thus, the emergence of novel variants with different characteristics should be examined further. On the other hand, an exposure-immunity trade-off could itself have evolutionary repercussions. For example, from an evolutionary perspective, lower transmissibility of infections in previously-exposed individuals could select against certain pathogen phenotypes. Thus, future work should use evolutionary-epidemiological models to untangle these potential evolutionary trajectories.

Third, we have focused on immuno-epidemiology and ignored individual decision-making dynamics. However, behavioral drivers can be important for emerging and circulating infectious diseases (e.g., refs. ^28,42^). For example, prior research has examined in detail individual decision-making with respect to vaccination^43,44^, adaptive behavior^45^, social distancing^33^, and social norms^46^. Recent work has also explored intervention adherence from a game-theoretic perspective^34^, and has examined long-term behavioral-epidemiological outcomes in a coupled model for individual NPI adherence^35^. Thus, future research should examine the effects of an immunity-exposure trade-off on potential long-term behavioral-epidemiological outcomes for NPI adherence.

Fourth, we have only considered susceptibility to, transmissibility of, and the duration of secondary infections. However, primary and secondary infections could lead to long-term complications, such as Long COVID (e.g., ref. ^47^), which could result in elevated mortality long after recovery. Recent findings have shown that such mortality in combination with reinfection can trigger endogenous periodicity^48^, and it would be valuable to explore the implications of an immunity-exposure trade-off in these contexts. Relatedly, we have ignored the effect of accumulating immunity after multiple infections^18^. In particular, we have assumed that tertiary infections and beyond are indistinguishable from secondary infections. In reality, these each could have their own characteristics, and incorporating these details in our framework would also be fruitful. Furthermore, we have assumed that the characteristics of secondary infections are bounded by those of primary infections. However, antibody-dependent enhancement could lead to a secondary infection that is worse than the primary infection encountered by a host (e.g., in dengue^49^), and future research should incorporate this in our modeling framework. Finally, more refined models should investigate the effects of severity-blocking immunity in detail (see e.g., ref. ^19^ for such a model), and its interplay with vaccination.

While we have examined the effects of vaccine durability, we have assumed for simplicity that the relationship between immunity and exposure is the same whether immune responses are the result of vaccination or recovery from infection. In reality, vaccines could lead to a different relationship between exposure and immunity, and dose-dependence in vaccinated individuals could also change with different vaccination formulations. These details should be explored in detail with refined analyses.

We have focused on the impacts of NPIs on the endemic equilibrium, and we have accomplished this by assuming that NPIs reduce the transmission rate. However, NPIs are likely to be implemented dynamically, and this may interfere with any underlying seasonal cycle. Furthermore, for emerging and re-emerging pathogens, understanding the impact of NPIs on transient dynamics and the underlying effects of host immune responses is crucial. To study these questions, future models should include these additional details.

To guide public health recommendations on long-term NPI usage, our work underlines the need to quantify the confluence of exposure with immuno-epidemiology and pathogen evolution. For example, it will be crucial to address whether this effect is restricted to infections like SARS-CoV-2, and if there are population heterogeneities in the strength of interaction. To accomplish this, cohort studies across ages and immunodominance should be initiated (e.g., as outlined in ref. ^36^). In particular, such studies could characterize multiple features of dose-dependent infections in previously exposed individuals. More broadly, our results highlight the importance of monitoring population susceptibility for circulating and emerging pathogens via sero-surveillance initiatives, such as a Global Immunological Observatory^50–52^.

## Supplementary information

Supplementary Information

Description of Additional Supplementary Files

Code to generate figures

## Data Availability

This paper contains no data.

## Code availability

A code file to reproduce all the figures is available as supplementary material.
