# Supplementary material for: Epidemiological impacts of nonpharmaceutical interventions are modulated by immunity exposure trade offs
Source: Commun Med (Lond). 2026 May 1;6:262. doi: 10.1038/s43856-026-01492-y (PMC13136377; doi:10.1038/s43856-026-01492-y)
Supplement: Supplementary file 1 — Supplementary Information [file 43856_2026_1492_MOESM1_ESM.pdf]

Supplementary Information for: "Epidemiological impacts of nonpharmaceutical interventions are modulated by immunity exposure trade offs"

Chadi M. Saad-Roy[1,2,3,4,5,*] Bjarke Frost Nielsen[6,7,8]
Margaret L. Lind[9,10] Caroline E. Wagner[11] Arne Traulsen[12]
C. Jessica E. Metcalf[6,13] Mike Boots[2,14] Derek A. T. Cummings[15,16]
Bryan T. Grenfell[6,13]

[1]Miller Institute for Basic Research in Science, University of California, Berkeley, USA
[2] Department of Integrative Biology, University of California, Berkeley, USA
[3] Department of Mathematics, University of British Columbia, Vancouver, Canada
[4] Department of Microbiology and Immunology, University of British Columbia, Vancouver, Canada
[5] Biodiversity Research Centre, University of British Columbia, Vancouver, BC, Canada
[6] Department of Ecology and Evolutionary Biology, Princeton University, Princeton, USA
[7] Niels Bohr Institute, University of Copenhagen, Copenhagen, Denmark
[8] PandemiX Center, Roskilde University, Roskilde, Denmark
[9] Department of Epidemiology of Microbial Diseases, Yale School of Public Health, New Haven, CT, USA
[10] Department of Epidemiology, Boston University School of Public Health, Boston, MA, USA
[11] Department of Bioengineering, McGill University, QC, Canada
[12] Max Planck Institute for Evolutionary Biology, Plön, Germany
[13] School of Public and International Affairs, Princeton University, Princeton, USA
[14] Department of Biosciences, University of Exeter, Exeter, UK
[15] Department of Biology, University of Florida, Gainesville, FL, USA
[16] Emerging Pathogens Institute, University of Florida, Gainesville, FL, USA
*chadi.saadroy@ubc.ca

Appendix

For the analyses below, we notice that p always multiplies β, and thus $p\beta$ can be treated as a unique parameter β. For clarity in the below calculations, we also denote δ_{vax} as δ_V.

Computing the basic reproduction number $\mathcal{R}_0$

In the absence of disease, $\widehat{I}_{P,0} = \widehat{I}_{S,0} = R_0 = 0$, and $S_{P,0} = \frac{\mu}{\mu+\nu}$, $S_{S,0} = \frac{\nu}{\mu+\nu}\frac{\delta_V}{\delta_V+\nu+\mu}$ and $V_0 = \frac{\nu}{\mu+\nu}\frac{\nu+\mu}{\delta_V+\nu+\mu}$. Using the I_P and I_S equations with the next-generation matrix [1, 2], it follows that

$$F = \begin{pmatrix} \beta S_{P,0} & \alpha\beta S_{P,0} \\ \varepsilon\beta S_{S,0} & \varepsilon\alpha\beta S_{S,0} \end{pmatrix}, \quad V = \begin{pmatrix} \gamma_P + \mu & 0 \\ 0 & \gamma_S + \mu \end{pmatrix}, \tag{1}$$

so that (where ρ denotes the spectral radius of the matrix)

$$\mathcal{R}_0 = \rho(FV^{-1}) = \rho\begin{pmatrix} \frac{\beta S_{P,0}}{\gamma_P+\mu} & \frac{\alpha\beta S_{P,0}}{\gamma_S+\mu} \\ \frac{\varepsilon\beta S_{S,0}}{\gamma_P+\mu} & \frac{\varepsilon\alpha\beta S_{S,0}}{\gamma_S+\mu} \end{pmatrix} = \frac{\beta S_{P,0}}{\gamma_P+\mu} + \varepsilon\alpha\frac{\beta S_{S,0}}{\gamma_S+\mu}. \tag{2}$$

Equilibria

Theorem 1. *Suppose $\gamma_S \geq \gamma_P$. If $\mathcal{R}_0 > 1$, there is a unique endemic equilibrium $\widehat{P}$. On the other hand, if $\mathcal{R}_0 < 1$, there is only the disease-free equilibrium P_0.*

Proof. Using the equations at equilibrium and defining $Y = I_P + \alpha I_S$, it follows that

$$\widehat{S}_P = \frac{\mu}{\mu+\nu+\beta\widehat{Y}}, \tag{3a}$$

$$\widehat{I}_P = \frac{\beta\widehat{Y}\widehat{S}_P}{\gamma_P+\mu} = \frac{\beta\widehat{Y}\mu}{(\gamma_P+\mu)(\mu+\nu+\beta\widehat{Y})}, \tag{3b}$$

$$\widehat{R} = \frac{\gamma_P\widehat{I}_P + \gamma_S\widehat{I}_S}{\mu+\delta}, \quad \widehat{V} = \frac{\nu(\widehat{S}_P+\widehat{S}_S)}{\mu+\delta_V}, \quad \widehat{I}_S = \frac{\varepsilon\beta\widehat{Y}\widehat{S}_S}{\gamma_S+\mu}, \tag{3c}$$

$$\widehat{S}_S = \frac{\delta\widehat{R}+\delta_V\widehat{V}}{\mu+\nu+\varepsilon\beta\widehat{Y}}, \quad \text{and} \quad \widehat{Y} = \widehat{I}_P + \alpha\widehat{I}_S. \tag{3d}$$

Substituting these in, we get that

$$\widehat{S}_S = \frac{1}{\mu + \nu + \varepsilon\beta\widehat{Y}} \left(\delta \frac{\gamma_P \widehat{I}_P + \gamma_S \frac{\varepsilon\beta\widehat{Y}\widehat{S}_S}{\gamma_S + \mu}}{\mu + \delta} + \frac{\delta_V}{\mu + \delta_V} \nu (\widehat{S}_P + \widehat{S}_S) \right). \tag{4}$$

Thus, it follows that

$$\widehat{S}_S \left(1 - \frac{\frac{\delta}{\delta+\mu}\frac{\gamma_S}{\gamma_S+\mu}\varepsilon\beta\widehat{Y} + \frac{\delta_V}{\mu+\delta_V}\nu}{\mu + \nu + \varepsilon\beta\widehat{Y}} \right) = \frac{\frac{\delta}{\delta+\mu}\gamma_P \widehat{I}_P + \frac{\delta_V}{\mu+\delta_V}\nu\widehat{S}_P}{\mu + \nu + \varepsilon\beta\widehat{Y}}. \tag{5}$$

Since $\nu - \frac{\delta_V}{\mu+\delta_V}\nu = \nu\frac{\mu}{\mu+\delta_V}$ and $\varepsilon\beta\widehat{Y}\left(1 - \frac{\delta}{\delta+\mu}\frac{\gamma_S}{\gamma_S+\mu}\right) = \frac{\mu(\mu+\delta+\gamma_S)}{(\delta+\mu)(\mu+\gamma_S)}\varepsilon\beta\widehat{Y}$, we find

$$\begin{aligned}
\widehat{S}_S &= \frac{1}{\mu} \frac{\frac{\delta}{\delta+\mu}\gamma_P \widehat{I}_P + \frac{\delta_V}{\mu+\delta_V}\nu\widehat{S}_P}{1 + \frac{\nu}{\mu+\delta_V} + \frac{\mu+\delta+\gamma_S}{(\delta+\mu)(\mu+\gamma_S)}\varepsilon\beta\widehat{Y}} \\
&= \frac{1}{\mu} \frac{\frac{\delta}{\delta+\mu}\gamma_P \frac{\beta\widehat{Y}\widehat{S}_P}{\gamma_P+\mu} + \frac{\delta_V}{\mu+\delta_V}\nu\widehat{S}_P}{1 + \frac{\nu}{\mu+\delta_V} + \frac{\mu+\delta+\gamma_S}{(\delta+\mu)(\mu+\gamma_S)}\varepsilon\beta\widehat{Y}} \\
&= \frac{1}{\mu + \nu + \beta\widehat{Y}} \frac{\frac{\delta}{\delta+\mu}\gamma_P \frac{\beta\widehat{Y}}{\gamma_P+\mu} + \frac{\delta_V}{\mu+\delta_V}\nu}{1 + \frac{\nu}{\mu+\delta_V} + \frac{\mu+\delta+\gamma_S}{(\delta+\mu)(\mu+\gamma_S)}\varepsilon\beta\widehat{Y}}.
\end{aligned} \tag{6}$$

Using the equation for $\widehat{Y}$, it follows that

$$\widehat{Y} = \frac{\beta\widehat{Y}}{\mu + \nu + \beta\widehat{Y}} \left(\frac{\mu}{\gamma_P + \mu} + \frac{\varepsilon\alpha}{\gamma_S + \mu} \left(\frac{\frac{\delta}{\delta+\mu}\frac{\gamma_P}{\gamma_P+\mu}\beta\widehat{Y} + \frac{\delta_V}{\mu+\delta_V}\nu}{1 + \frac{\nu}{\mu+\delta_V} + \frac{\mu+\delta+\gamma_S}{(\delta+\mu)(\mu+\gamma_S)}\varepsilon\beta\widehat{Y}} \right) \right). \tag{7}$$

Thus,

$$\begin{aligned}
&(\mu + \nu + \beta\widehat{Y}) \left(1 + \frac{\nu}{\mu + \delta_V} + \frac{\mu + \delta + \gamma_S}{(\delta + \mu)(\mu + \gamma_S)}\varepsilon\beta\widehat{Y} \right) \\
&= \mu\frac{\beta}{\gamma_P + \mu}\left(1 + \frac{\nu}{\mu + \delta_V} + \frac{\mu + \delta + \gamma_S}{(\delta + \mu)(\mu + \gamma_S)}\varepsilon\beta\widehat{Y}\right) + \varepsilon\alpha\frac{\beta}{\gamma_S + \mu}\left(\frac{\delta}{\delta + \mu}\frac{\gamma_P}{\gamma_P + \mu}\beta\widehat{Y} + \frac{\delta_V}{\mu + \delta_V}\nu\right).
\end{aligned} \tag{8}$$

Therefore, subtracting the RHS from both sides gives that

$$f(\widehat{Y}) = A\widehat{Y}^2 + B\widehat{Y} + C = 0, \tag{9}$$

where

$$A = \beta\varepsilon\beta\frac{\mu+\delta+\gamma_S}{(\delta+\mu)(\mu+\gamma_S)}, \tag{10a}$$

$$\begin{aligned} C &= (\mu+\nu)\left(1+\frac{\nu}{\mu+\delta_V}\right) - \left(\mu\frac{\beta}{\gamma_P+\mu}\left(1+\frac{\nu}{\mu+\delta_V}\right) + \varepsilon\alpha\frac{\beta}{\gamma_S+\mu}\frac{\delta_V}{\mu+\delta_V}\nu\right) \\ &= (\mu+\nu)\left(\frac{\mu+\nu+\delta_V}{\mu+\delta_V}\right)\left(1-\left(\frac{\mu}{\mu+\nu}\frac{\beta}{\gamma_P+\mu} + \varepsilon\alpha\frac{\beta}{\gamma_S+\mu}\frac{\delta_V}{\mu+\nu+\delta_V}\frac{\nu}{\mu+\nu}\right)\right) \\ &= (\mu+\nu)\left(\frac{\mu+\nu+\delta_V}{\mu+\delta_V}\right)(1-\mathcal{R}_0). \end{aligned} \tag{10b}$$

Thus, $C < 0$ if $\mathcal{R}_0 > 1$ and $C > 0$ if $\mathcal{R}_0 < 1$.

Suppose first that $\varepsilon > 0$, which means $A > 0$. Since $A > 0$, it follows by Descartes' Rule of Signs that there is a unique positive root if $\mathcal{R}_0 > 1$.

Computing B gives

$$\begin{aligned} B &= \beta + \beta\frac{\nu}{\mu+\delta_V} + (\mu+\nu)\varepsilon\beta\frac{\mu+\delta+\gamma_S}{(\delta+\mu)(\mu+\gamma_S)} \\ &\quad - \mu\frac{\beta}{\gamma_P+\mu}\varepsilon\beta\frac{\mu+\delta+\gamma_S}{(\delta+\mu)(\mu+\gamma_S)} - \varepsilon\alpha\frac{\beta}{\gamma_S+\mu}\frac{\delta}{\delta+\mu}\frac{\gamma_P}{\gamma_P+\mu}\beta \\ &= (\mu+\nu)\varepsilon\beta\frac{\mu+\delta+\gamma_S}{(\delta+\mu)(\mu+\gamma_S)}\left(1-\frac{\mu}{\mu+\nu}\frac{\beta}{\gamma_P+\mu}\right) \\ &\quad + \beta\frac{\nu+\mu+\delta_V}{\mu+\delta_V}\left(1-\varepsilon\alpha\frac{\beta}{\gamma_S+\mu}\frac{\delta}{\delta+\mu}\frac{\gamma_P}{\gamma_P+\mu}\frac{\mu+\delta_V}{\nu+\mu+\delta_V}\right), \end{aligned} \tag{11}$$

and so $\widehat{Y}$ is the positive solution to the quadratic equation when $\varepsilon > 0$, and $\widehat{Y} = -\frac{C}{B}$ if $\varepsilon = 0$.

It then follows that

$$\widehat{I}_S = \frac{1}{\alpha}\left(\widehat{Y} - \frac{\beta\widehat{Y}\mu}{(\gamma_P+\mu)(\mu+\nu+\beta\widehat{Y})}\right). \tag{12}$$

Since $\gamma_S \geq \gamma_P$ by assumption of the theorem, it follows that $\mathcal{R}_0 \geq \varepsilon\alpha\frac{\beta}{\gamma_S+\mu}\left(\frac{\mu}{\mu+\nu} + \frac{\nu}{\mu+\nu}\frac{\delta_V}{\delta_V+\mu+\nu}\right) = \varepsilon\alpha\frac{\beta}{\gamma_S+\mu}\left(\frac{\delta_V+\mu}{\delta_V+\mu+\nu}\right)$. Furthermore, note that $\mathcal{R}_0 \geq \frac{\beta}{\gamma_P+\mu}\frac{\mu}{\mu+\nu}$. Thus, if $\mathcal{R}_0 < 1$, it follows that $B > 0$. Then, since $A \geq 0$, $B > 0$, and $C > 0$, $f(\widehat{Y}) = 0$ has no positive roots if $\mathcal{R}_0 < 1$. Therefore, if $\mathcal{R}_0 < 1$, the disease-free equilibrium is the only equilibrium.

□

Remark 1. *Consider the case where there are two non-interacting groups of size* N_1 *and* N_2*, where each group has a different immunity-exposure trade-off. Since the groups are non-interacting, the population-level outcome will be a weighted sum of the individual group outcomes, scaled by the relative population sizes. Thus, our framework applies directly in this case.*

References

[1] Diekmann O, Heesterbeek JAP, Metz JAJ (1990) On the definition and the computation of the basic reproduction ratio R_0 in models for infectious diseases in heterogeneous populations. *Journal of Mathematical Biology* 28(4):365–382.

[2] van den Driessche P, Watmough J (2002) Reproduction numbers and sub-threshold endemic equilibria for compartmental models of disease transmission. *Mathematical Biosciences* 180(1):29 – 48.

Supplementary Figures

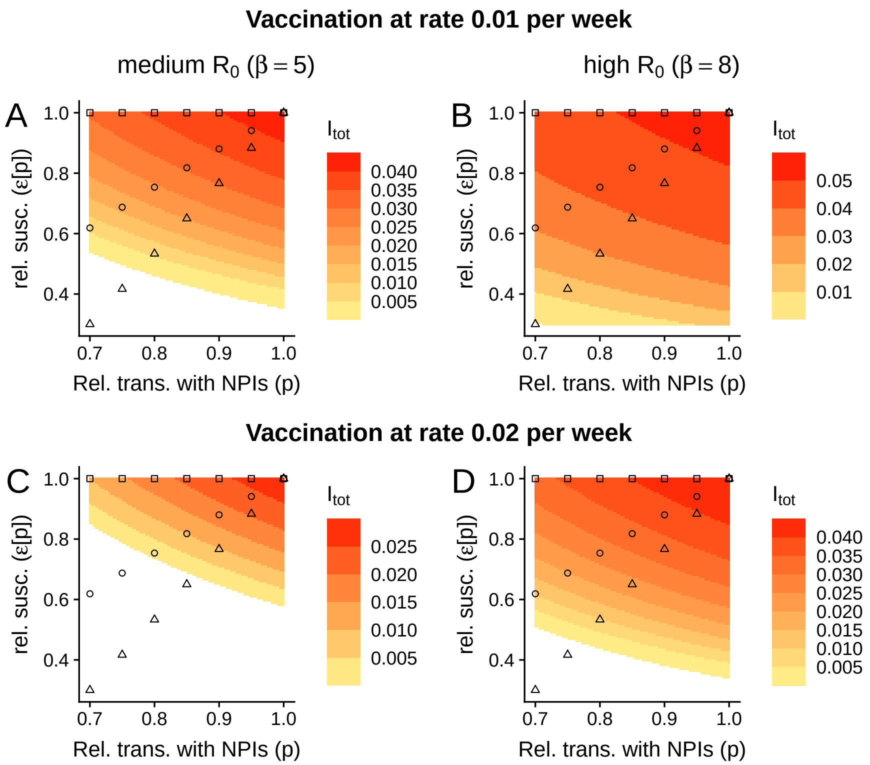

Figure S1: Effects of a broadly-protective vaccine with complete immunity for an average of two years (*i.e.*, $\frac{1}{\delta_{\text{vax}}} = 2$ years). All details and other parameters are as in the corresponding panels of Figure 3.

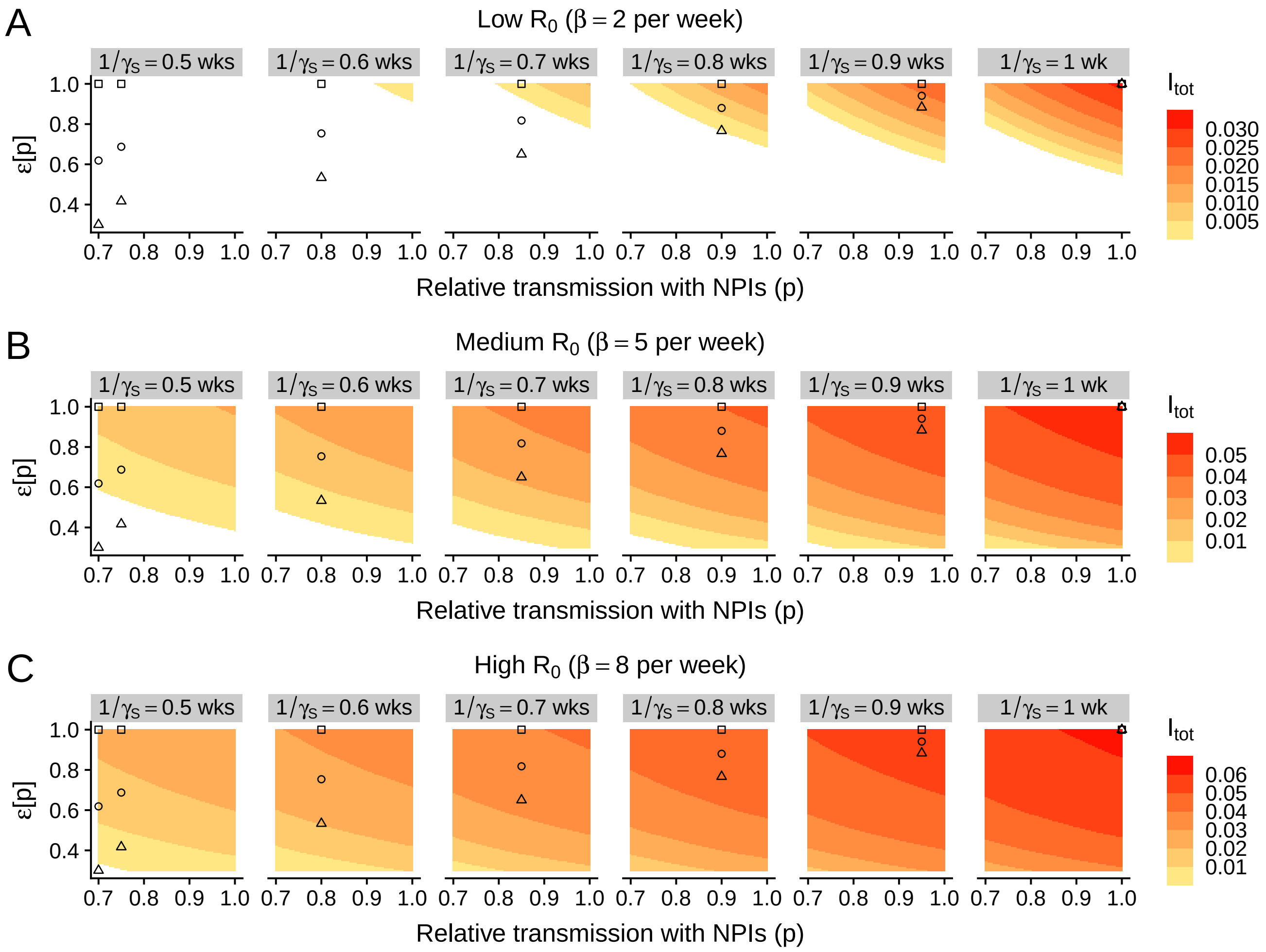

Figure S2: Effects of vaccination (at rate $\nu = 0.01$) on the equilibrium level of infections when immunity trades off with relative susceptibility to secondary infection and duration of secondary infection. All details and other parameters for panels (A)–(C) are as Figures 4A–4C, respectively.

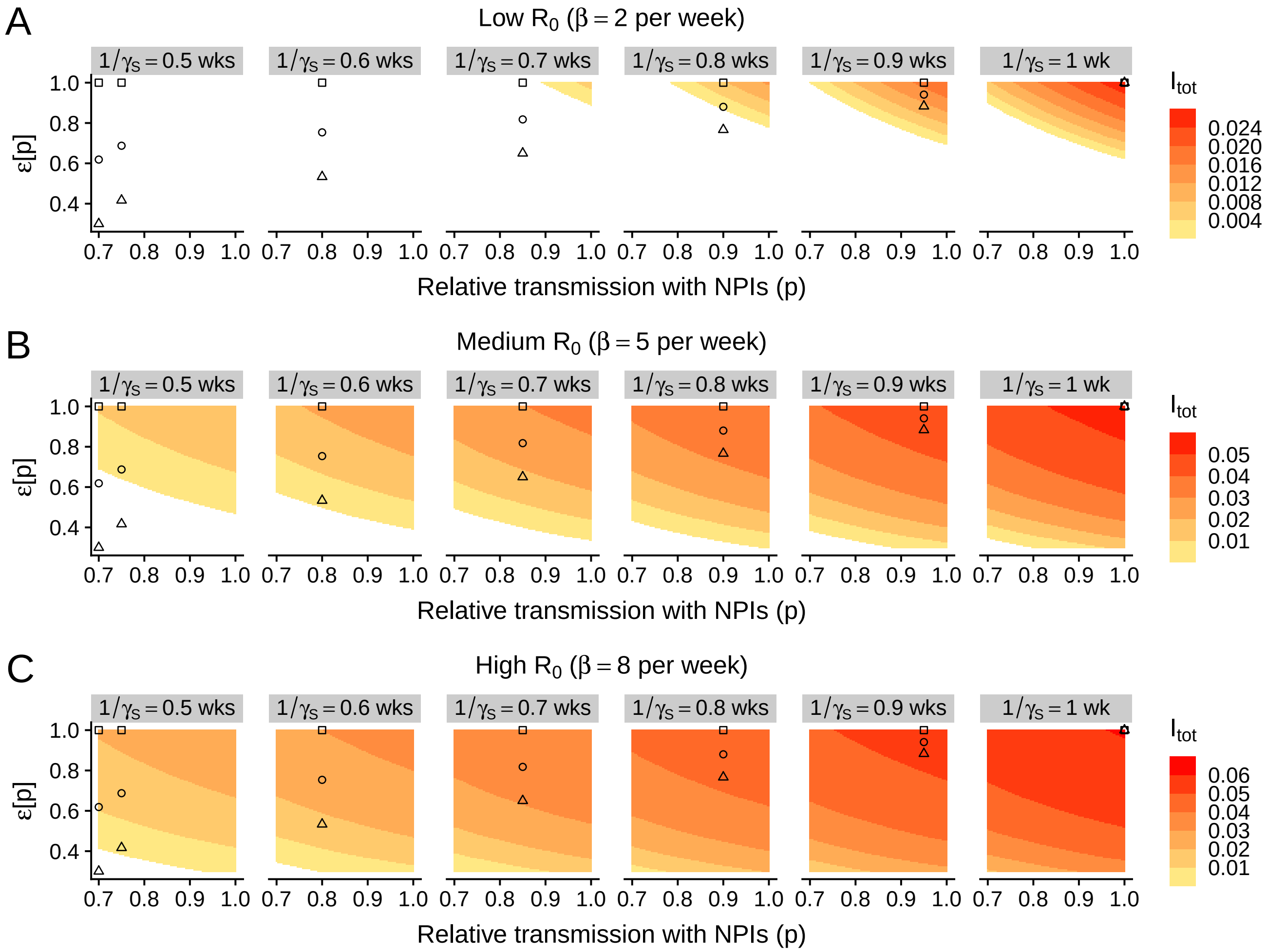

Figure S3: Impacts of elevated vaccination rates when the duration of secondary infection also decreases with decreasing exposure. All details and parameters are as in Figure S2, except that $\nu = 0.02$ instead.

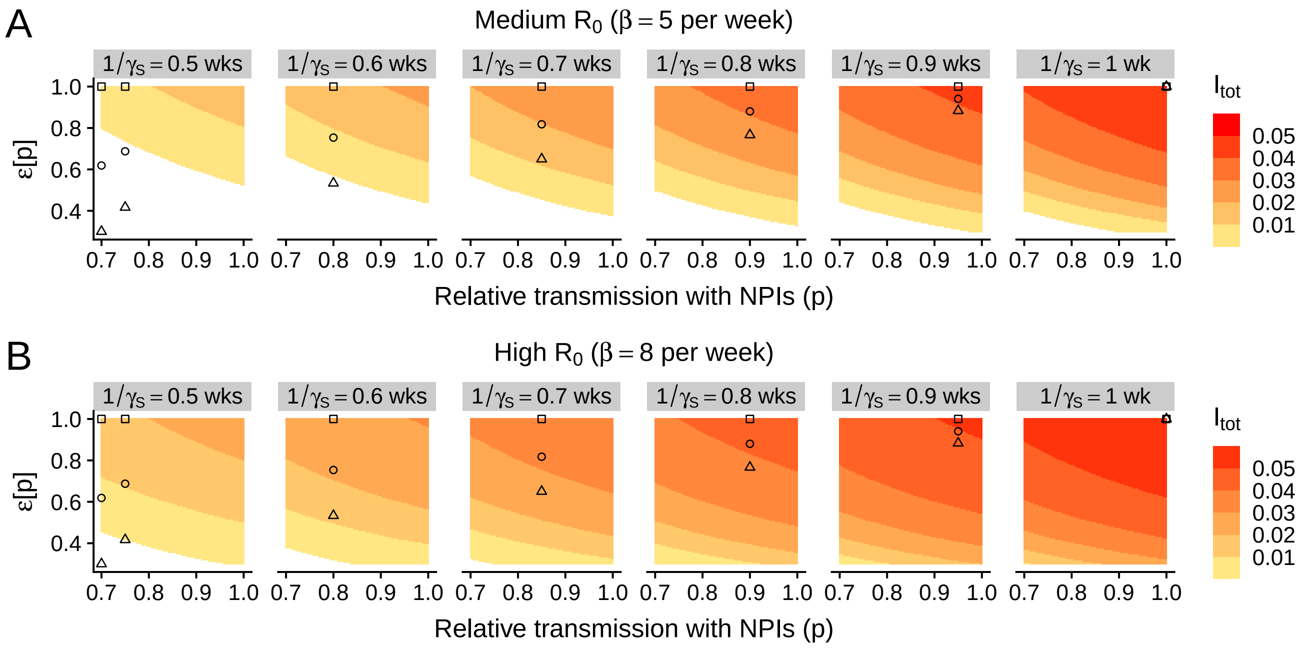

Figure S4: Effects of a broadly-protective vaccine with complete immunity for an average of a year (*i.e.*, $\frac{1}{\delta_{\text{vax}}} = 1$ year) with $\nu = 0.01$ per week, when immunity trades off with relative susceptibility of secondary infections and duration of secondary infection. All details and other parameters for panels (*A*) and (*B*) are as in Figures 4B–4C, respectively.

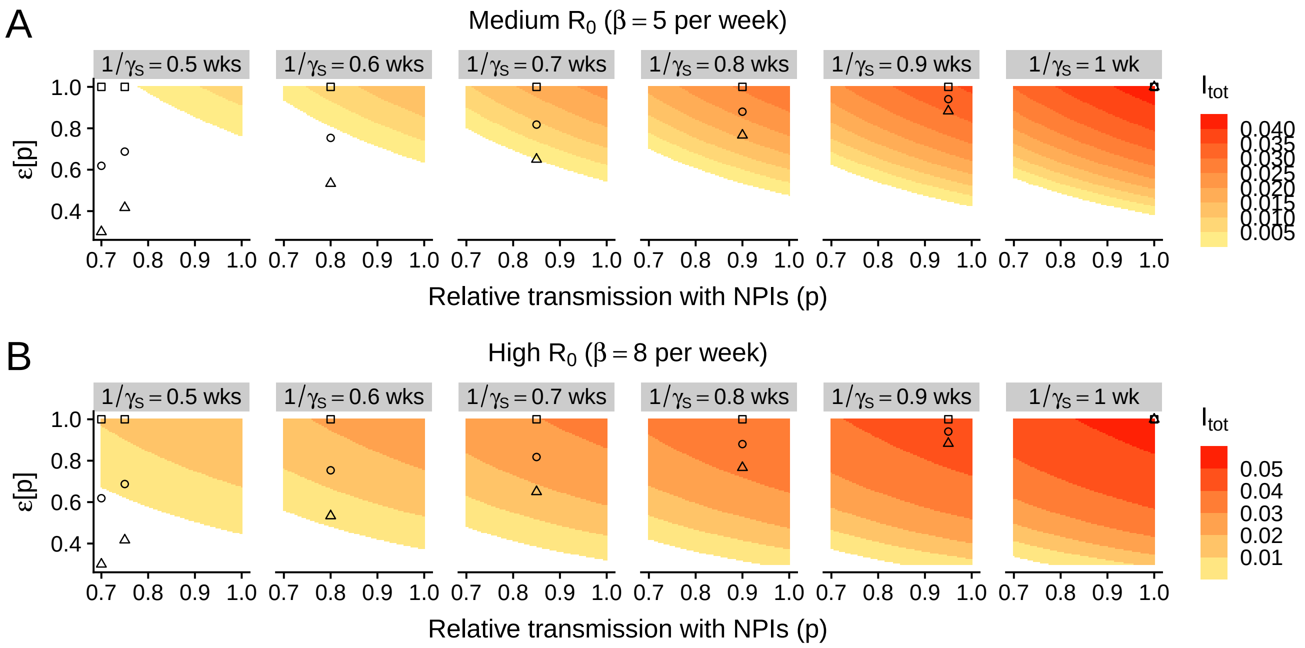

Figure S5: As in Figure S4, but with a vaccination rate $\nu = 0.02$ per week.

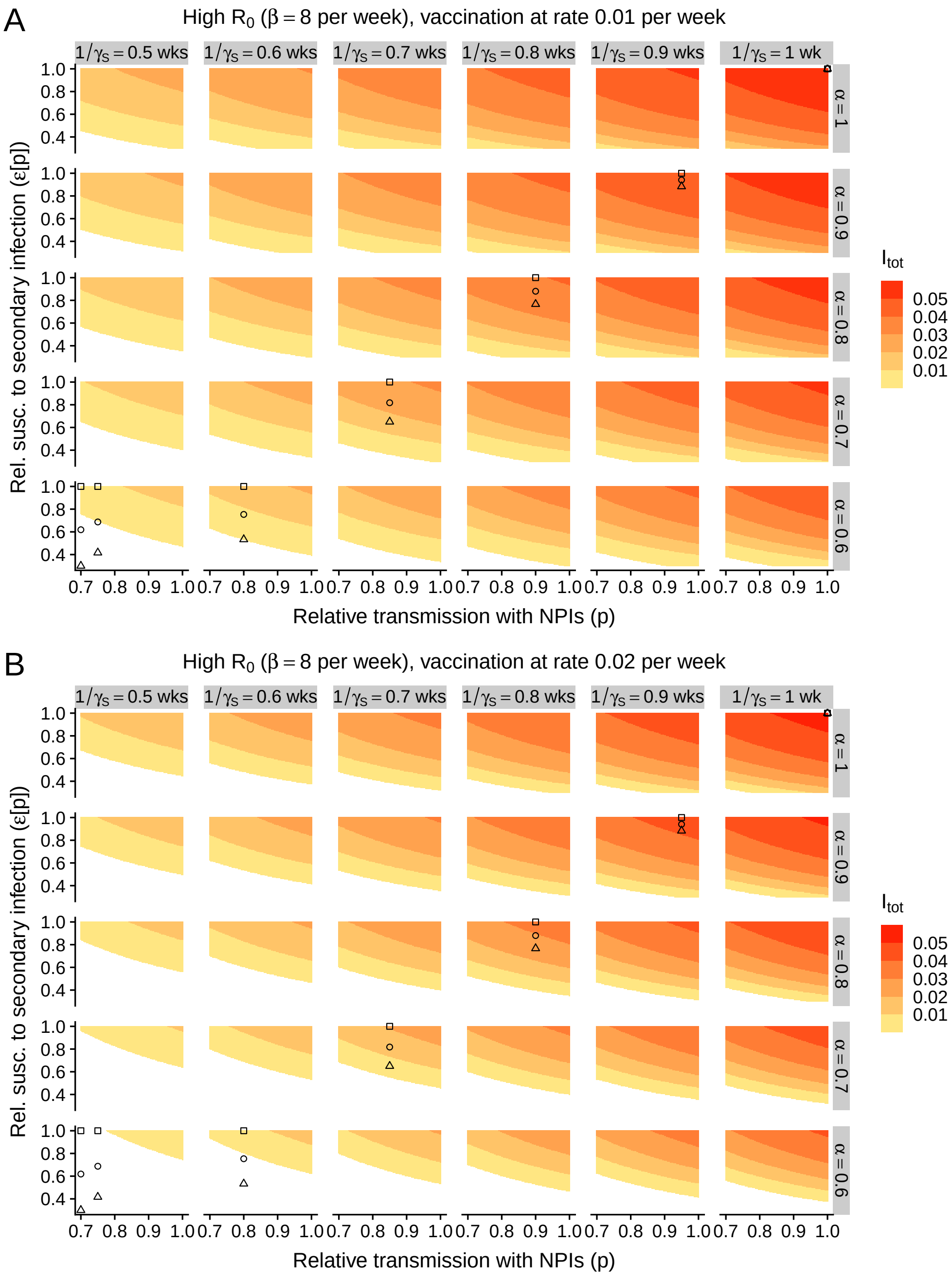

Figure S6: Effects of a broadly-protective vaccine with complete immunity for an average of one year (*i.e.* $\frac{1}{\delta_{\text{vax}}} = 1$ year), when the relative susceptibility to secondary infection, the duration of secondary infections, and the relative transmissibility of secondary infections are all potentially dose-dependent. Here, vaccination rates are (*A*) $\nu = 0.01$ and (*B*) $\nu = 0.02$. All details and other parameters are as in Figures 4D and 4E.

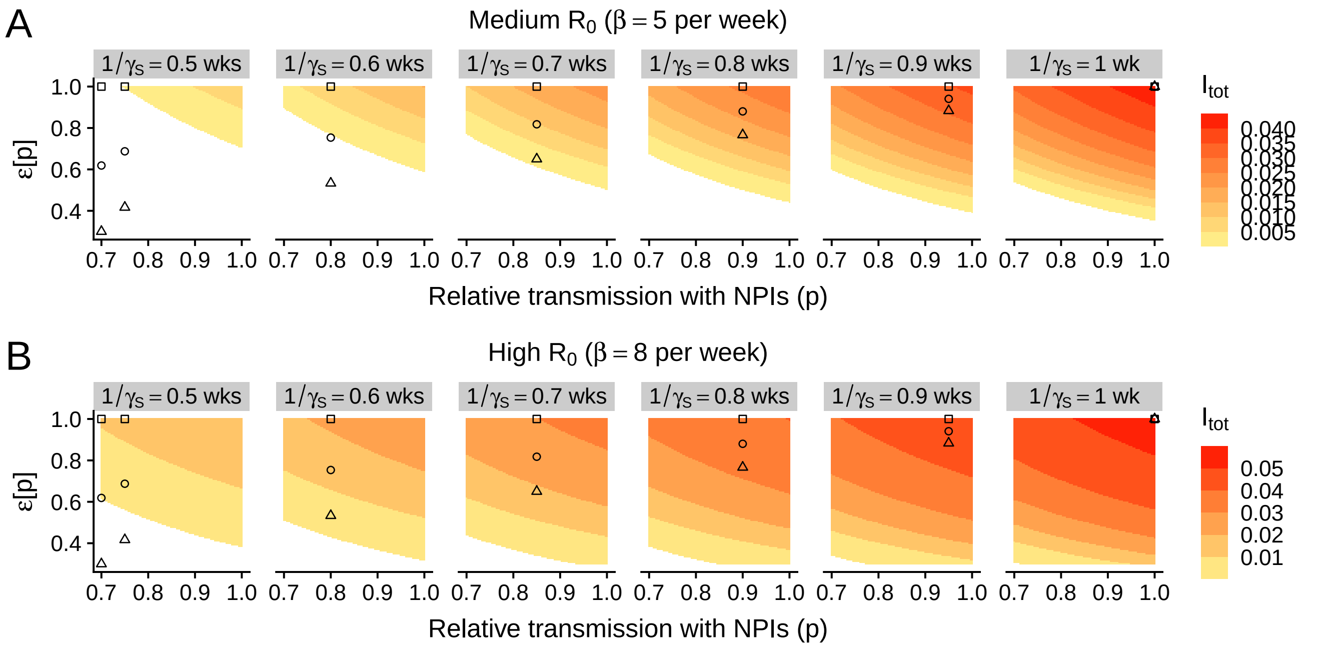

Figure S7: Effects of a broadly-protective vaccine that imparts complete immunity for an average of two years (*i.e.* $\frac{1}{\delta_{\text{vax}}} = 2$ years) with $\nu = 0.01$, when both the relative susceptibility to secondary infection and the duration of secondary infections are exposure-dependent. All details and other parameters for panels (*A*) and (*B*) are as in Figures 4B–4C, respectively.

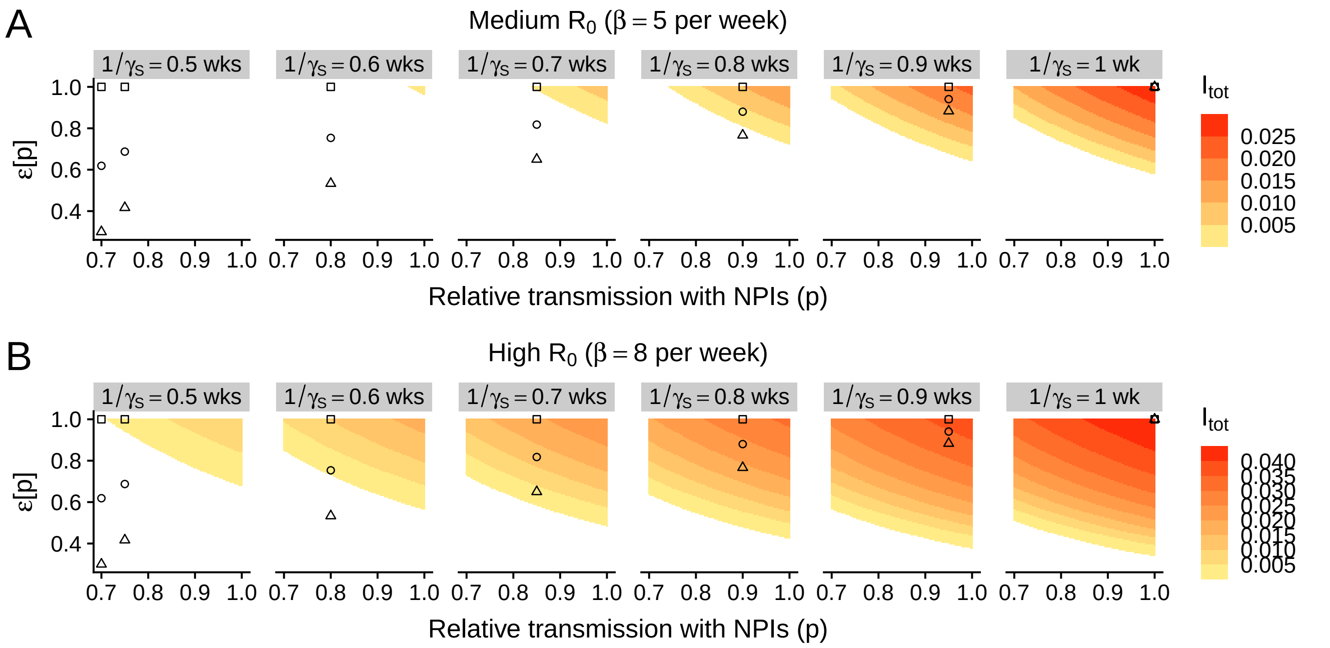

Figure S8: As in Figure S7, but with $\nu = 0.02$ per week.

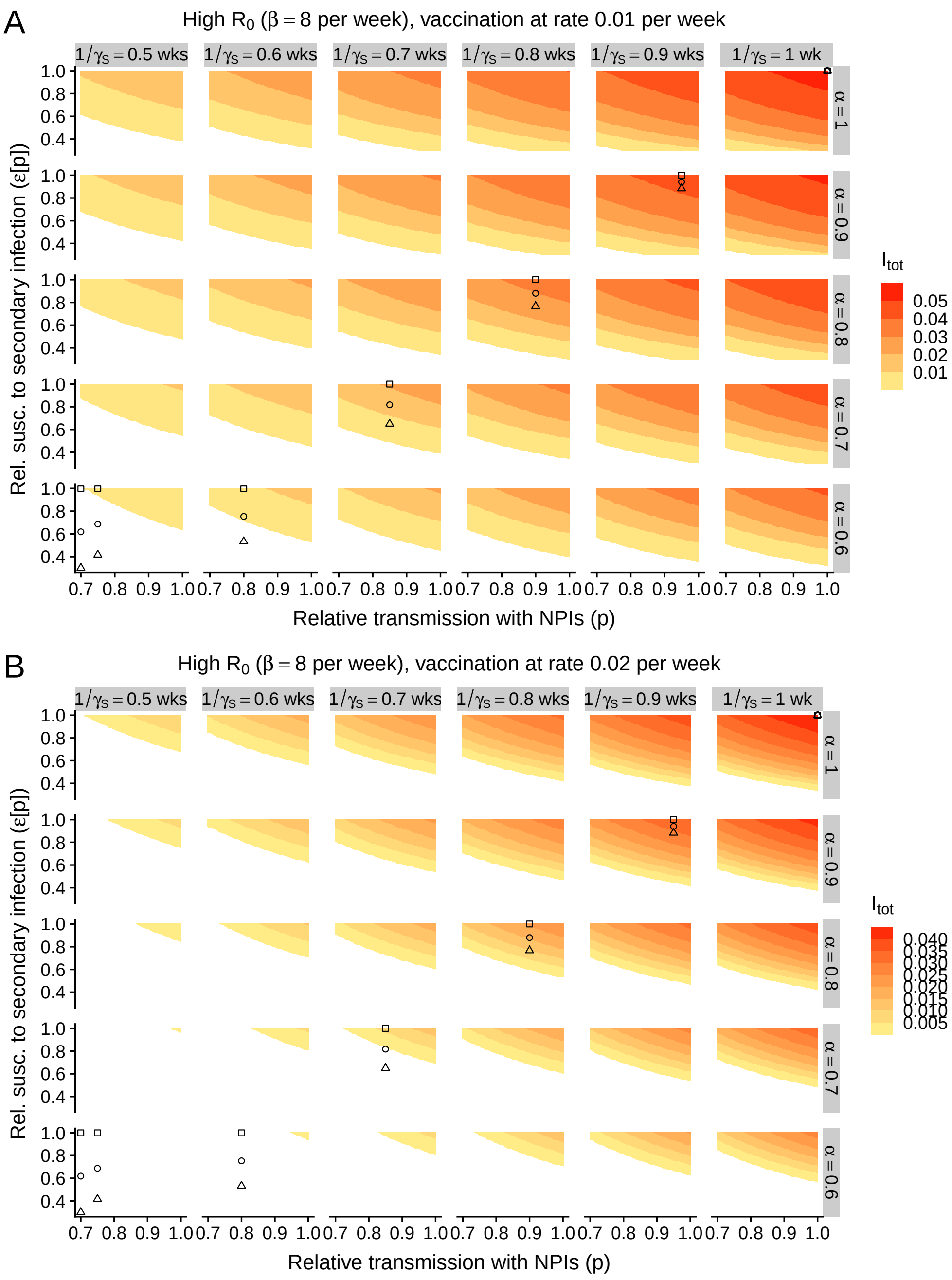

Figure S9: Effects of an even more broadly protective vaccine than in Figure S5, where complete immunity is imparted for on average 2 years after vaccination (*i.e.*, $\frac{1}{\delta_{\text{vax}}} = 2$ years), and with (*A*) $\nu = 0.01$ and (*B*) $\nu = 0.02$. All other parameters are as in Figures 4D and 4E.
