# Supplementary material for: Epidemiological impacts of nonpharmaceutical interventions are modulated by immunity exposure trade offs
Source: Commun Med (Lond). 2026 May 1;6:262. doi: 10.1038/s43856-026-01492-y (PMC13136377; doi:10.1038/s43856-026-01492-y)
Supplement: Supplementary file 2 — Description of Additional Supplementary Files [file 43856_2026_1492_MOESM2_ESM.docx]

**Description of Additional Supplementary Files**

File name: Supplementary Data

Description: A code file to reproduce all the figures
